# Advances in the dissection of *Anopheles*–*Plasmodium* interactions

**DOI:** 10.1371/journal.ppat.1012965

**Published:** 2025-03-31

**Authors:** Sally A. Saab, Victor Cardoso-Jaime, Mary Kefi, George Dimopoulos

**Affiliations:** W. Harry Feinstone Department of Molecular Microbiology and Immunology, Bloomberg School of Public Health, Johns Hopkins University, Baltimore, Maryland, United States America; National Institutes of Health, UNITED STATES OF AMERICA

## Abstract

Malaria is a life-threatening mosquito-borne disease caused by the *Plasmodium* parasite, responsible for more than half a million deaths annually and principally involving children. The successful transmission of malaria by *Anopheles* mosquitoes relies on complex successive interactions between the parasite and various mosquito organs, host factors, and restriction factors. This review summarizes our current understanding of the mechanisms regulating *Plasmodium* infection of the mosquito vector at successive plasmodial developmental stages and highlights potential transmission-blocking targets and strategies.

## 1. Introduction

Mosquitoes are vectors for a number of human pathogens that cause severe disease, such as malaria, Zika, chikungunya, dengue, and yellow fever [1]. According to the most recent World Health Organization (WHO) report, malaria caused an estimated 249 million cases and 608,000 deaths worldwide in 2022, representing an increase of 5 million cases over 2021 numbers [2]. Six species of the *Plasmodium* protozoan parasite have been shown to cause human malaria: *Plasmodium falciparum, P. vivax, P. ovale, P. malariae, P. knowlesi,* and *P. cynomologi* [3]. In Africa, *P. falciparum* is the most commonly fatal species and is mainly transmitted by adult female members of the *Anopheles gambiae* species complex [4]. This complex comprises eight morphologically identical sibling species that vary in their geographical prevalence [5–7]. Apart from the invertebrate mosquito vector, the life cycle of *Plasmodium* also involves the vertebrate human host; however, all of the previous attempts that have focused on the human host have failed to produce a vaccine that can effectively eradicate malaria [8]. Despite the progression to advanced-clinical trials, most available vaccines cannot be considered sufficient as a stand-alone measure to worldwide malaria eradication [9]. Moreover, emerging parasite resistance against the anti-malarial drugs jeopardizes their efficiency [10, 11]. For instance, the emerging *Plasmodium* resistance to the fast-acting antimalarial drug artemisinin has led to a significant reduction in the drug’s efficacy, resulting in lower treatment success rates and prolonged illness [12]. Thus, control efforts have also been focused on transmission-blocking strategies that are designed to prevent the parasite from infecting the mosquito vector. One promising strategy is based on generating genetically engineered mosquitoes resistant to *Plasmodium* infection [13], a strategy that requires extensive knowledge of both mosquito and parasite biology. Here, we review our current understanding of *Anopheles–Plasmodium* interactions and potential manipulations to block the mosquito’s vectorial capacity.

## 2. *Plasmodium* development in the lumen of the mosquito gut

### 2.1. Gametogenesis

The sexual reproductive phase of the *Plasmodium* parasite begins when the mosquito ingests, along with mosquito saliva, an infected gametocyte-containing blood meal from a vertebrate host. Once the ingested gametocytes reach the mosquito midgut, they are exposed to the mosquito-derived byproduct of the tryptophan degradation, xanthurenic acid (XA), along with a temperature drop and pH elevation, and they respond by differentiating into mature gametes in a process known as gametogenesis [14–16]. Gametogenesis starts with the rounding up of the parasites within the ingested erythrocytes, preceding the “inside-out” egress of the parasites into the mosquito gut. This egress involves a sequential membrane rupture that starts with the rupture of the inner parasitophorous vacuole membrane and is followed by the rupture of the external erythrocyte membrane [17,18].

Gametogenesis differs between male and female gametocytes. Recent transcriptomic studies have reported that the histone variant H3.3 protein, which is enriched in female gametocytes, is responsible for the repression of the male-specific genes to favor female properties [19]. Male gametogenesis, which is known as exflagellation, involves three rapid consecutive DNA replication cycles that give rise to eight motile microgametes within 15 min after activation [18,20]; in contrast, female gametogenesis results in a single non-motile female macrogamete that is ready for fertilization [21].

A well-orchestrated molecular mechanism occurs in *Plasmodium* gametocytes to initiate gametogenesis. XA-mediated boosting of guanylyl cyclase (GC) activity increases the level of the second messenger 3′−5′-cyclic guanosine monophosphate (cGMP) in the gametocyte [22]. Despite the presence of two membrane GC proteins (GCα and GCβ) in *P. falciparum* [23], only GCα seems to be involved in cGMP production during gametogenesis, since disrupting GCβ does not prevent XA-stimulated gamete formation [24]. Recent studies in *Plasmodium yoelii* using CRISPR/Cas9 genome editing have shown that this XA-stimulated cGMP production is mediated by a membrane protein that spans multiple membranes. Known as gametogenesis essential protein 1 (GEP1), this protein interacts with GCα, enhancing its activity [25]. Another membrane protein known as G-protein-coupled receptor 180 (GPR180) promotes this cGMP elevation in *P. berghei* [26]. Protein kinase G (PKG) responds to the elevated levels of cGMP by phosphorylating a multipass membrane protein, recently named important for Ca^2+^ mobilization 1 (ICM1), which stimulates the mobilization of stored calcium into the cytosol [27–29]. This increase in cytosolic Ca^2+^ activates several calcium-dependent protein kinases (CDPK), which in turn translate this signal into various cellular responses required for gametogenesis. For instance, CDPK1 in *P. falciparum* is critical for both male and female gametogenesis. In the absence of CDPK1, female gametocytes are incapable of rounding up post-activation, as opposed to male gametocytes, which can round up but do not engage in exflagellation [30]. On the other hand, CDPK2 is only essential during male exflagellation [31]. Another major CDPK in male gametogenesis is CDPK4, which was recently shown to be responsible for essential processes, including DNA replication, mRNA translation, and cell motility [32].

In addition to CDPKs, other families of protein kinases are also implicated in *Plasmodium* male gametogenesis. The male-specific mitogen-activated protein kinase 2 (MAP-2) in *P. falciparum* has been reported to be essential for proper axonemal beating [33]. Recently, cyclin-dependent kinase-related kinases (CRKs) have also been found to be critical for male exflagellation, with deletion of CRK5 in *P. falciparum* resulting in defective male gametogenesis [34].

### 2.2. Gamete fertilization

**Fig 1:**
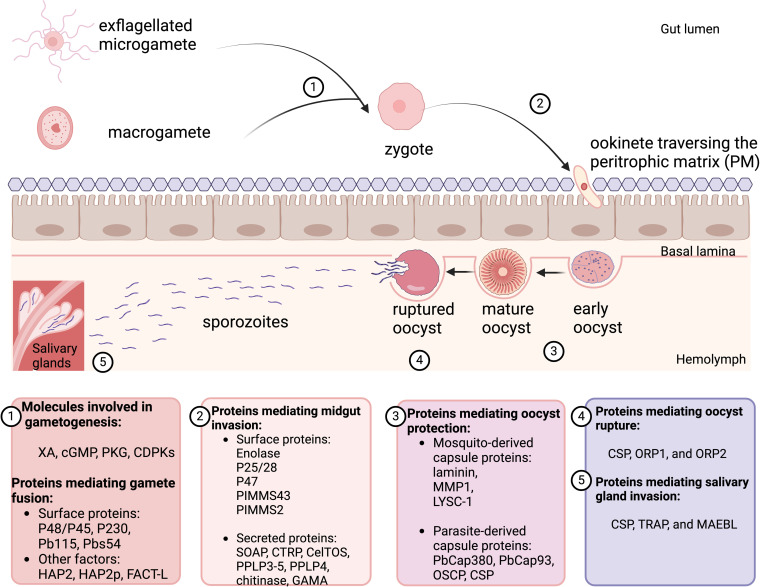
The *Plasmodium* life cycle in the mosquito vector. Following an infected blood meal, the ingested *Plasmodium* gametocytes develop into male and female gametes that can fuse together to form a zygote. The zygote then differentiates into a motile ookinete and invades the midgut epithelium after traversing the peritrophic matrix separating the ingested blood meal from the epithelial cells. Right after it reaches the extracellular space between the epithelium and the basal lamina, the ookinete develops into an early oocyst, which in turn undergoes sporogony to give rise to a mature oocyst holding thousands of sporozoites. Following rupture of the oocyst, the released sporozoites invade the salivary glands and are ready for transmission to a new host. Proteins mediating the various phases of the life cycle are mentioned in the panels below the corresponding developmental stages. Created in BioRender. Saab, S. (2025) https://BioRender.com/p65s417.

After their egress from the ingested erythrocytes, *Plasmodium* male and female gametes fuse to form a diploid zygote within the first hour after intake of a blood meal [20,35]. Several surface proteins present on both male and female gametes are involved in this process (Fig 1). Two gamete proteins, P48/45 and P230, of the six-cysteine protein family form a complex on the surface of the gamete that is required for male fertility and subsequent fusion with the female macrogamete in *P. falciparum* [36,37]. Another surface six-cysteine protein, known as P47, is solely expressed on the female macrogamete surface and is necessary for female fertility in *P. berghei* but not *P. falciparum* [38,39]. Moreover, deleting the highly conserved hapless 2 protein (HAP2, also known as generative cell-specific 1) in *P. berghei* male microgametes does not prevent the male and female gametes from adhering to each other, but it inhibits their fusion [40]. In addition to HAP2, HAP2 paralog (HAP2p) is essential for the fertilization of *P. falciparum* gametes. Deleting either of these two membrane fusogens, which are located throughout the flagella of the male microgametes, will prevent gamete fertilization [41]. In addition, the histone chaperone protein FACT-L (named after the large subunit of the human protein, facilitates chromatin transcription [FACT]) plays a crucial role in male gamete fertility and subsequent gamete fusion, but the mechanism by which this nuclear protein affects fertilization is still not clear [42]. Recent evidence underlines the requirement for additional membrane proteins, Pb115 and Pbs54, for the attachment of male and female gametes in *P. berghei* [43,44].

Most of the previously mentioned *Plasmodium* surface proteins have been studied for their transmission-blocking vaccine (TBV) potential, and continuous efforts have been made to identify various domains in P230, P48/45, P47, and HAP2 that could be used for inducing transmission-reducing antibodies in the host [45–50].

### 2.3. Development of the zygote into a motile ookinete

Following gamete fusion, the diploid zygote undergoes DNA replication, resulting in a tetraploid phase that persists up to the formation of the haploid sporozoites within the oocysts. This process has been shown to be regulated by the NIMA-related kinases Nek-2 and Nek-4, which in turn are essential for ookinete differentiation [51].

At 18–30 h after a blood meal, the zygote differentiates into a motile ookinete stage, which invades the midgut epithelium of the mosquito after traversing the peritrophic matrix, the semipermeable chitinous layer separating the blood bolus from the midgut epithelial cells (Fig 1) [52–54]. Studies of the dynamics of zygote-ookinete development in *An. coluzzii* mosquitoes by live immunofluorescence microscopy have demonstrated that *P. falciparum* passes through several intermediate stages before becoming a mature ookinete. Formation of mature ookinetes peaks at 23 h following the ingestion of infected blood and persists in the gut up to 36 hpost-infection [55].

Recent single-cell RNA-seq data have linked these various stages to their gene expression profiles, with the essential genes for DNA replication and metabolism detected in early zygotes, and those required for host invasion identified in later ookinete stages [56]. Also, studies in *P. berghei* have shown that only the maternally inherited alleles are active in the zygote-ookinete stages; the paternally inherited genes are expressed later on, during midgut invasion [57]. Such gene expression profiles are translated into several cell structure modifications, including the establishment of a special organelle known as the inner membrane complex (IMC), located beneath the *Plasmodium* cell membrane, as well as the cell polarization that occurs during zygote-to-ookinete differentiation [58].

While the transition between the various *Plasmodium* developmental stages is strongly coordinated by various apicomplexan Apetala-2 transcription factors [59–61], translational regulation has also been reported to play an essential role in repressing the expression of certain mRNAs [62]. Recently, Hirai *et al*. have identified an RNA binding protein in *P. berghei* known as Pb103, which has the two zinc finger domains required for zygote differentiation, most probably through translational repression [63]. In addition to translational regulation, post-translational modifications are also crucial for zygote differentiation. Studies using *P. yoelii* have pointed to several pellicle proteins, including IMC subcompartment proteins 1 and 3 (ISP1 and ISP3), IMC‐residing palmitoyl‐S‐acyl‐transferase (PAT) DHHC2, and the cytoskeletal microtubule β-tubulin, being involved in the proper arrangement of the cytoskeletal subpellicular microtubules through a process that requires palmitoylation to promote zygote elongation [64]. Thus, inhibiting palmitoylation might be a promising way to prevent *Plasmodium* development [65].

### 2.4. Anti-plasmodial immune responses in the lumen of the mosquito midgut

Upon the ingestion of an infected blood meal, the mosquito gut microbiota, mainly composed of gram-negative bacteria [66,67], proliferate and play a protective role against the *Plasmodium* parasite by immune priming and the secretion of factors that can inhibit parasite development. Hence, elimination of the microbiota in antibiotic-treated *An. gambiae* mosquitoes leads to a significant increase in *P. falciparum* oocyst numbers, thus rendering the mosquitoes more susceptible to the parasite [66]. Accordingly, an *Enterobacter* species isolated from *Anopheles arabiensis* in Zambia can directly kill *Plasmodium* through the production of reactive oxygen species (ROS) [68]. Furthermore, the *Chromobacterium* species, which was initially isolated from *Aedes aegypti* mosquitoes in Panama, exerts an anti-*Plasmodium* effect by secreting romidepsin, a histone deacetylase inhibitor, which inhibits *Plasmodium* [69,70]. In addition, *Serratia marcescens* bacteria isolated from mosquito midguts also secrete uncharacterized metabolites that can directly inhibit the *Plasmodium* ookinete stage, independent of the mosquito vector [71]. Recently, certain strains of *Serratia ureilytica*, isolated from wild *An. sinensis* in Tengchong, China, have been shown to produce an anti-plasmodial lipase that can target the early gametocyte stage of the parasite in the mosquito midgut, thus preventing ookinete formation [72].

Apart from their direct effects, the midgut microbiota can also indirectly protect a host against *Plasmodium* by triggering the expression of the mosquito immune genes. After a blood meal, the gut microbiota proliferates and releases immune elicitors that can activate the immune deficiency (Imd) pathway, which targets the *P. falciparum* ookinete stage through multiple effectors [73–76]. The activation of the Imd signaling pathway results in the nuclear translocation of the NF-κB transcription factor Rel2, triggering the expression of several immune genes, including anti-*Plasmodium* factors and AMPs [74]. Defensins, cecropins, attacin, and gambicin are the four different classes of AMPs identified in the *An. gambiae* genome [77]. These AMPs target a wide range of pathogens, including *Plasmodium*, gram-positive and gram-negative bacteria, fungi, and yeasts [74]. The Imd pathway acts in concert with the Toll signaling pathway, contributing to the expression of defensin 1, cecropin 1, and gambicin [78]. While activating the Imd pathway abolishes *P. falciparum* infection in three different *Anopheles* species, it has no effect on *P. berghei*, highlighting the specificity of various immune pathways for various pathogens [75]. Furthermore, the antiparasitic role of the Imd signaling pathway has been further emphasized in genetically modified *An. stephensi* overexpressing Rel2 in both the midgut and the fat bodies, which renders the mosquitoes resistant to *P. falciparum* [79].

Recently, DNA methylation was shown to play a crucial role in regulating the midgut anti-plasmodial immune responses driving the differential susceptibility of various *Anopheles albimanus* phenotypes to *P. berghei* [80]. Interestingly, the transgenic depletion of several microRNAs in *An. gambiae* using microRNA sponges modulates the expression of the midgut immune genes, making the mosquitoes more resistant to parasitic infection [81].

## 3. *Plasmodium* ookinete invasion of the mosquito midgut epithelium

### 3.1. Ookinete invasion of the mosquito gut

The ookinete first crosses the chitin-containing peritrophic matrix by releasing chitinase and then invades the epithelial cells through a complex process that requires several interactions between the parasite and the mosquito factors [82] (Fig 1). The epithelial cell invasion is mediated in part by enolase on the ookinete surface, which interacts with the ingested plasminogen and the epithelial cells, allowing midgut invasion to occur [83]. Other ookinete-surface proteins also facilitate the traversal of the midgut epithelium. For example, P25/28, P47, and *Plasmodium* infection of the mosquito midgut screen 43 (PIMMS43) help the ookinete evade the mosquito’s immune responses. At the same time, PIMMS2 is essential for midgut invasion [84–87].

Apart from the proteins expressed on its surface, mature ookinetes also secrete other micronemal proteins that mediate midgut invasion. These proteins include the secreted ookinete adhesive protein (SOAP), circumsporozoite- and TRAP-related protein (CTRP), cell-traversal protein for ookinete and sporozoites (CelTOS), *Plasmodium* perforin-like protein 3-5 (PPLP3-5), chitinase, and GPI-anchored micronemal antigen (GAMA) [88–95]. In addition, the A domain of CTRP is crucial for the ookinete’s gliding motility, which in turn makes possible the penetration of the mosquito midgut [96].

Recently, another member of the perforin-like proteins, PPLP4, was also reported to have a role in traversing the epithelia [97]. Along with the parasite proteins, peritrophic matrix-associated fibrinogen-related protein 1 (FREP1), which is released by the epithelial cells, can directly bind to ookinetes, enabling them to penetrate both the peritrophic matrix and the epithelium [98]. The fibrinogen-like domain of FREP1, which is responsible for its direct interaction with *Plasmodium* gametocytes and ookinetes, is highly conserved between the various *Anopheles* species and is an effective TBV candidate [99,100].

Recently, *Plasmodium* α-tubulin-1, expressed on the apical end of the ookinetes, was shown to interact with FREP1 to direct the ookinetes toward the peritrophic matrix, thus facilitating parasite invasion [101]. Blocking the direct interaction between FREP1 and the parasite through anti-fibrinogen-like domain or anti-α-tubulin-1 antibodies significantly reduces *P. falciparum* infection in *An. gambiae* mosquitoes, making the FREP1-tubulin interaction a robust transmission-blocking target [99,101]. Interestingly, *An*. *gambiae* alanyl aminopeptidase N1 (AgAPN1), which is expressed on midgut epithelial cells, can also play an essential role in facilitating the invasion by *P. berghei* and *P. falciparum* ookinetes [102]. Despite this significant research, more work is still needed to elucidate more fully the interactions between AgAPN1 and the parasite; however, determining the crystal structure of AgAPN1 has been of great help in working out how to target this enzyme with domain-specific antibodies that are promising for transmission-blocking [103,104].

Ookinetes have been suggested to penetrate the midgut epithelium through both intercellular and intracellular routes by using their ability to move by gliding. Use of either of these invasion routes was recently shown to be a mosquito species-specific process [105]. Studies with *P. yoelii* have linked this gliding motility to the IMC sub-compartment protein 1 [ISP1]-mediated polarization of guanylate cyclase β from the cytoplasm to the “ookinete extrados site”, a site posterior to the apical structure in mature ookinetes, thereby activating the cGMP-dependent PKG signaling pathway that is responsible for the gliding motility [106].

While in the ookinete stage, the parasite passes through a major population bottleneck, making this stage a target for future antimalarial transmission-blocking interventions [107]. Surface or secreted ookinete proteins that are involved in motility and invasion are robust candidates for TBVs [108,109]. In addition to vaccines, transmission-blocking strategies also include the generation of transgenic mosquitoes engineered to block the parasite and prevent its invasion [110]. For instance, transgenic *An. stephensi* expressing exogenous single-chain antibodies (scFv) that act against chitinase or Pf25, along with the sporozoite circumsporozoite protein (CSP), can successfully clear *P. falciparum* sporozoites from mosquitoes [111]. Also, CRISPR/Cas9-mediated FREP1 knockout in *An. gambiae* suppresses both human and rodent parasite infection [112].

### 3.2. Immune responses within the epithelial cells

Despite its ability to digest and penetrate the peritrophic matrix, the parasite remains protected in part from epithelium-elicited immune responses, thanks to cross-linkage of the mucus layer proteins by immunomodulatory peroxidase and the intestinal dual oxidase [113]. Another redox system mediates a more robust anti-plasmodial immune response, known as midgut-epithelial cell nitration, that can further modify the ookinete and make it a target for the complement system [114].

Nitration is a two-step reaction involving the synthesis of nitric oxide (NO), followed by a peroxidase reaction [115]. The synthesis of NO after *Plasmodium* infection requires the upregulation of nitric oxide synthase (NOS) downstream of the JAK-STAT signaling pathway. NOS plays a critical role in reducing *Plasmodium* oocyst survival [116]. Also, a heme peroxidase (HPX2)/NADPH oxidase 5 (NOX5) system, induced by the Jun-N-terminal kinase (JNK) signaling pathway, is responsible for mediating epithelial nitration and subsequent ookinete surface modification. These reactions mark the ookinete for targeting by the mosquito’s complement-like system through recruitment of the complement-like thioester-containing protein 1 (TEP1) as the ookinetes reach the extracellular space between the epithelium and the basal lamina and become exposed to the hemolymph [114,117]. Certain Pfs47 variants specific for the African *Plasmodium* strains can suppress midgut epithelial nitration in *An. gambiae* mosquitoes through binding to a certain Pfs47 midgut receptor (P47Rec), thereby enabling evasion of the mosquito’s immune response [118–120]. Recent studies addressing the mechanistic function of P47Rec have revealed the involvement of heat shock protein 70 cognate 3 (Hsc70-3) in inhibiting the caspase-mediated apoptosis of the invaded midgut cells and therefore disrupting epithelial nitration [121].

### 3.3. Hemolymph immune responses against the ookinete stage

**Fig 2:**
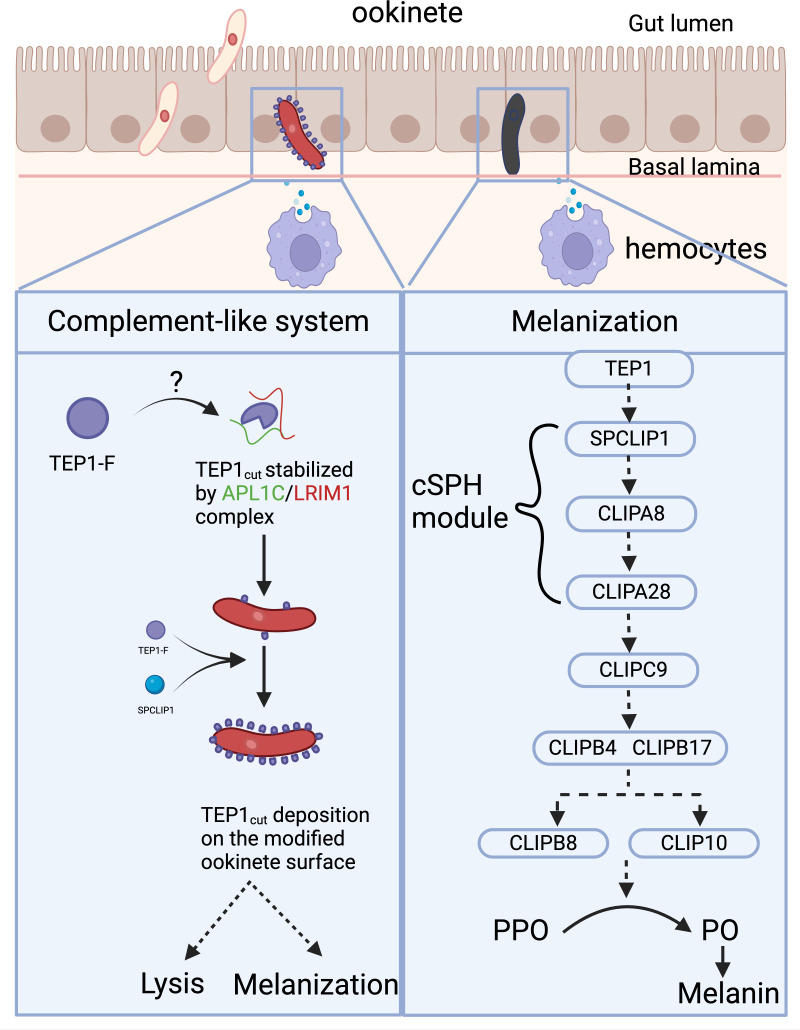
Mosquito immune responses against the early ookinete stage. *Plasmodium* ookinete midgut invasion induces nitration, modifying the parasite surface for TEP1 recruitment. TEP1 is secreted into the hemolymph in its full form (TEP1-F). Upon activation, TEP1-F is cleaved by an unknown factor into the active cut form (TEP1_cut_), which is stabilized in the hemolymph by binding to the APL1C/LRIM1 heterodimer. Recognition of the ookinete results in the deposition and accumulation of TEP1 _cut_ on the parasite surface which is promoted by SPCLIP1 due to the cleavage of more TEP1-F by an unknown TEP1 convertase. TEP1-marked ookinetes are then targeted for lysis or, in rare cases, melanization, which mainly occurs in certain refractory backgrounds (left panel). Melanization requires a cascade of catalytic and noncatalytic CLIP serine proteases that regulate the cleavage of PPO zymogen into active PO, the key protein in melanin synthesis. Altogether, thus far, TEP1 seems to be the factor farthest upstream, followed by the core cSPH module (SPCLIP1-CLIPA8-CLIPA28) that acts upstream of the catalytic CLIPC9. Downstream of the CLIPC9 comes CLIPBs, with CLIPB4 and CLIPB17 seeming to be the farthest upstream; the cascade then bifurcates into two different branches: one converging on CLIPB8 and the other on CLIPB10. Dashed arrows indicate that the steps require further characterization (right panel). Created in BioRender. Saab, S. (2025) https://BioRender.com/r16n889.

After bypassing the local immune responses induced in the midgut, ookinetes become exposed to systemic immune responses on the basal side of the epithelium that are triggered in the fat body and hemocytes [122]. For instance, abdominal tissues such as the fat body can respond to the diffusion of NO and hydrogen peroxide from the mosquito midgut to the hemolymph by releasing anti-plasmodial AMPs and other factors [123]. Hemocytes can also be activated, after the nitration of infected epithelial cells, to release vesicles containing unknown factors that promote the binding of TEP1 to the ookinete [124], targeting the parasite for elimination through either lysis or melanization (Fig 2) [125,126].

Although TEP1 expression was first attributed to hemocytes, recent studies have suggested that other organs, such as the fat body, are also involved in this process [127,128]. After the full-length TEP1-F is secreted, a portion of it is cleaved into TEP1_cut_ to become activated [129]. TEP1_cut_ is then stabilized in the hemolymph by a disulfide-linked heterodimer made up of two leucine-rich repeat proteins (LRRs), leucine-rich repeat immune protein 1 (LRIM1) and *Anopheles Plasmodium*-responsive leucine-rich repeat 1 (APL1C) (Fig 2). Interestingly, a knockdown of LRIM1/APL1C not only results in a higher number of oocysts in the midgut but also in a nonspecific deposition of TEP1 _cut_ on mosquito tissues, suggesting the participation of LRIM1/APL1C in avoiding an autoimmune response [130–133]. APL1 is encoded by a family of three genes, APL1A, APL1B, and APL1C. APL1A is induced by the Imd pathway and is *P. falciparum*-specific; in contrast, APL1C is induced by the Toll pathway and is *P. berghei*-specific [134,135]. APL1C acts against both extracellular ookinetes and circulating sporozoites of rodent *Plasmodium*, but not human [136].

TEP1 function is further promoted by the non-catalytic CLIP serine protease (cSPH) known as SPCLIP1, which mediates the accumulation of TEP1_cut_ on the microbial surface, thereby triggering lysis or melanization [137]. Another cSPH, CLIPA2, has also been shown to negatively regulate TEP1 activity by preventing its cleavage into TEP1_cut_ [138]. It is well established that the complement-like system plays a crucial role in parasite elimination; however, the process by which the TEP1/LRIM/APL1C complex induces pathogen lysis has not been fully dissected and warrants further investigation.

In addition to TEP1-mediated lysis, the melanization reaction observed in certain refractory genetic backgrounds makes *An. gambiae* mosquitoes highly resistant to *Plasmodium* ookinetes [139–141]. The deposition of melanin on the surface of a pathogen depends on a series of biochemical reactions that require active phenoloxidases (POs), which are released into the hemolymph as prophenoloxidase (PPO) zymogens. PPO cleavage is regulated by a cascade of CLIP domain serine proteases, which represent a large family of five subgroups (A to E) in *An. gambiae* mosquitoes [142,143]. Previous genetic studies have identified several cSPHs of subfamily A as playing a major nonredundant role in *P. berghei* melanization. For instance, SPCLIP1 (CLIPA30), CLIPA8, and CLIPA28 form a core cSPH module that positively regulates *P. berghei* ookinete melanization [137,144,145]. Such cSPHs are activated in an ordered manner downstream of TEP1, with SPCLIP1 being the farthest upstream, followed by CLIPA8 and then CLIPA28 [145]. Two CLIP-domain serine proteases, CLIPA2 and CLIPA14, have been shown to act as negative regulators of the melanization response [138,146]. In addition to cSPHs, several catalytic CLIP serine proteases of subfamilies B and C have also been identified as playing an essential role in the melanization response in *An. gambiae* mosquitoes. Individually silencing CLIPB4, CLIPB8, CLIPB10, CLIPB14, CLIPB17, or CLIPC9 partially reverses *P. berghei* melanization in refractory *An. gambiae* mosquitoes [144,147–149]. Recent *in vivo* studies have shown redundancy in the antimicrobial function of CLIPBs, which can be partially explained by a bifurcation of the cascade downstream of CLIPB4 and CLIPB17 into two branches, one converging on CLIPB8 and the other on CLIPB10 (Fig 2) [150]. Based on their capacity to cleave *Manduca sexta* PPOs *in vitro*, CLIPB4, CLIPB9, and CLIPB10 have been classified as prophenoloxidase-activating proteins (PAPs), which are inhibited by serine protease inhibitor 2 (serpin 2 or SRPN2) [148,151,152]. The core cSPH module acts upstream of the catalytic proteases, despite the cleavage of CLIPA8 by several recombinant CLIPBs *in vitro* [150], highlighting the complexity of this network.

Conversely, the C-type lectin 4 (CTL4) and MA2 (CTL2) play regulatory roles in protecting the parasite from melanization. For instance, silencing CTL4 in *An. gambiae* triggers TEP1-mediated melanization of *P. berghei* ookinetes [140,153,154], and CRISPR/Cas9-based CTL4 knockout *An. gambiae* mosquitoes show an enhanced ability to melanize *P. falciparum* ookinetes in a TEP1-independent manner [141]. Injecting recombinant CLT4/CTLMA2 into CTL4-silenced *An. gambiae* mosquitoes reversed the increase in the PO activity, suggesting a common role for the CLT4/CTLMA2 heterodimer in the melanization response [155]. The mechanisms governing CTL4-mediated protection of the ookinete from melanization and how cSPHs intervene to regulate PPO cleavage are still unclear and require further study.

A recent single-cell transcriptomic study in *An. gambiae* has demonstrated that the CLIP-domain serine proteases, CTL4/CTLMA2 and LRIM1, which are involved in the regulation of melanization, are highly expressed in the fat body, but PPOs are exclusively produced by hemocytes [156], suggesting that the mechanisms limiting *Plasmodium* infection also involve a systemic immune response.

Apart from the humoral immune responses, hemocytes are also critical for killing malaria parasites such as *P. berghei* that infect rodents. Hemocyte depletion in *An. gambiae* drastically increases the number of *P. berghei* parasites in the mosquito [127]. In addition to TEP1, hemocytes can also express other factors that either facilitate or limit *Plasmodium* development [156–160]. For example, a functional assay performed by Lombardo et al. [160] has shown that *An. gambiae* hemocytes express genes that function as *P. berghei* agonists, including a putative LRR protein, lipopolysaccharide-induced tumor necrosis factor alpha factor-like 6 (LL6), laminin A homolog, peptidase and trypsin-like domain-containing transmembrane protein, and a vesicular-type ATPase. They also express *P. berghei* antagonists, including von Willebrand factor-type A domain protein, collagen type IV protein, hexamerin 2 beta homolog, fibrinogen-related FBN8, and a CLIPB [161]. Smith and colleagues have reported similar findings in their proteomic analysis of *An. gambiae*, which revealed that phagocytic hemocytes play a dual role in *P. falciparum* infections by expressing anti-plasmodial immune effectors such as TEP1, defensin1, HPX2, and two components of the Ras family, the small GTPases Ras-related and Ras-homolog family member A, as well as *Plasmodium* protective factors such as lysozyme c-1 (LYSC1), scavenger-receptors SCRASP1 and SCRBQ2, LRIM15, and pretaporter (Prtp) [162]. Interestingly, several members of the fibrinogen-related proteins (FREP or FBN) that are mainly abundant in the hemolymph, including FBN8, FBN9, FBN30, FBN39, act as *Plasmodium* antagonists [161,163–165].

## 4. *Plasmodium* development in the hemolymph and hemolymph-triggered immune responses

### 4.1. Oocyst development and sporogony

After reaching the extracellular space separating the midgut epithelial cells from the basal lamina, the ookinete rounds up and develops into an oocyst, which in turn undergoes extensive mitotic divisions known as sporogony [166]. The mechanism triggering the developmental progression from ookinete to oocyst is still unclear; however, several mosquito and *Plasmodium* genes have been shown to be involved (Fig 1). Recently, three novel ookinete-expressed genes, PIMMS01, PIMMS57, and PIMMS22, were found to be critically involved in the development of the oocyst from the ookinete stage [167]. Moreover, the oocyst capsule also includes several mosquito-derived proteins, including laminin, matrix metalloprotease 1 (MMP1), and lysozyme c-1 (LYSC1), that assist the oocyst in hiding from the mosquito’s immune responses, thus favoring oocyst development [168]. Other parasite-derived proteins in the oocyst capsule, including the *P. berghei* oocyst capsule protein 380 (PbCap380), oocyst capsule-associated protein 93 (PbCap93), and the ookinete surface and oocyst capsule protein (OSCP), are essential for oocyst development and maintenance, and knocking out the genes encoding such proteins results in a reduced oocyst number [169–171].

Interestingly, a proteomic study has revealed unique, temporally regulated signatures in early, mid, and late oocysts in the rodent parasite [172]. For instance, expression of CSP as an oocyst capsule component after the onset of sporogony in rodent parasites is necessary for its escape from the melanization immune response in *An. stephensi* mosquitoes [173]. Sporogony eventually produces oocysts containing thousands of sporozoites by 14 days after the intake of a blood meal [168]. Recently, adipokinetic hormone signaling has been shown to be involved in facilitating *P. falciparum* sporogony [174]. Live fluorescence imaging of oocysts undergoing sporogony has revealed that sporozoite release is preceded by capsule thinning and small opening formation, thereby facilitating the sporozoites’ egress [175]. Although several proteins, including parasite-derived CSP and oocyst rupture protein (ORP) 1 and 2 [176], have been reported to be required for the oocyst rupture, the molecular mechanism driving this capsule thinning and the consequent sporozoite excystation remain poorly understood. Residence in the mosquito vector in limited numbers for about 2 weeks, along with their immobility, render the oocyst stage an appropriate transgenic target for blocking parasite transmission, but little is yet known about anti-oocyst effectors.

### 4.2. Immune responses against oocysts

**Fig 3:**
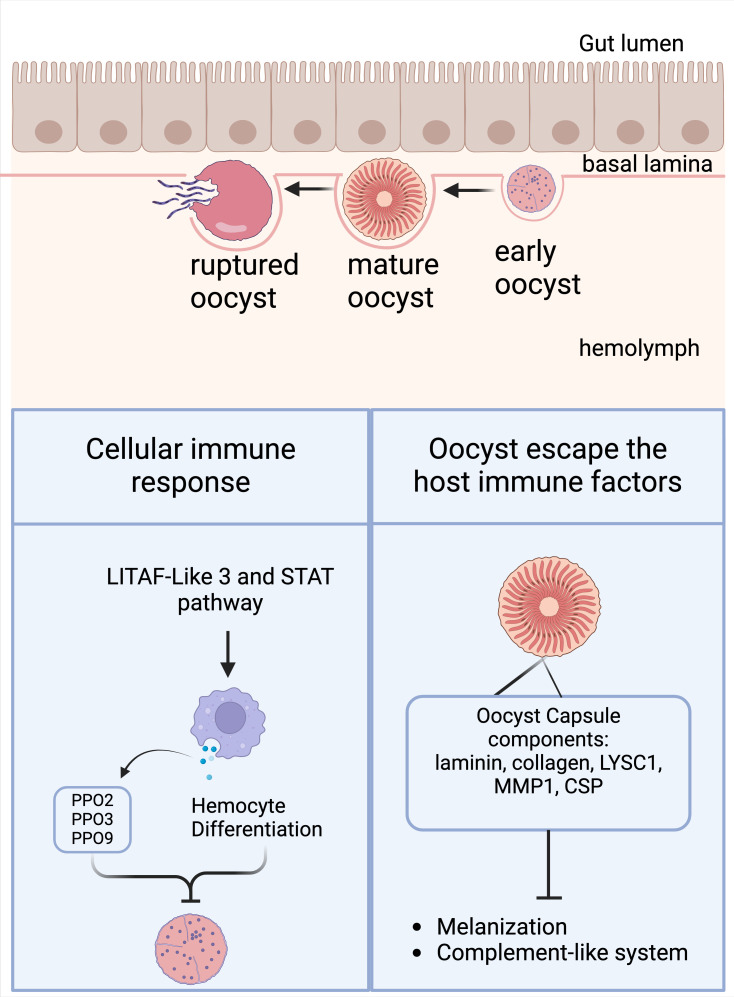
Mosquito late-phase immune responses against the oocyst stage. The main cellular immune response known to be triggered against the oocyst stage involves hemocyte differentiation driven by the action of the LITAF-Like 3 and STAT pathway. Hemocytes are the unique suppliers of PPOs, and PPO2, PPO3, and PPO9 limit oocyst survival without triggering melanin deposition (left panel). However, the oocyst stage can evade most of the hemolymph-mediated immune responses by exploiting its capsule components, such as laminin, collagen, LYSC1, MMP1, and CSP (right panel). Created in BioRender. Saab, S. (2025) https://BioRender.com/q60q481.

Since they reside between the midgut epithelial cells and the basal lamina for about 2 weeks, oocysts become extensively exposed to the immune factors that are involved in ookinete killing. However, oocysts seem to be the most resilient parasite stage in the mosquito [168,176]. Recently, oocysts of *P. yoelii* were shown to evade *An. stephensi* immune responses through the circumsporozoite protein (CSP). CSP disruption makes the parasite visible to the immune system through midgut nitration and hemocyte recruitment, with subsequent activation of the TOLL signaling pathway and oocyst TEP1-dependent melanization (Fig 3) [173]. Interestingly, in *An. gambiae*, PPO2, PPO3, and PPO9 limit the survival of *P. berghei* oocysts without triggering melanization, hinting at alternative functions that are not related to the classical roles of POs [127]. Recently, the oocyst stage has been shown to also be susceptible to the melanization immune response, in which co-silencing CLIPA2 and CLIPA14, the negative regulators of melanization, triggers the melanization of both *P. berghei* and *P. falciparum* oocysts [177]. Despite the fact that only late-stage *P. berghei* oocysts were melanized, this was not the case for *P. falciparum*, in which both early and late oocysts were found to be melanized [177].

Other studies have indicated that the STAT pathway and LITAF-like 3 limit early *P. berghei* and *P. falciparum* oocyst development through processes involving midgut and carcass nitration along with hemocyte differentiation (Fig 3) [116,178]. Interestingly, a unique stem-cell mediated response downstream of the JAK-STAT pathway has been shown to be responsible for eliminating oocysts [179]. Taken together, these studies suggest the involvement of systemic immune responses in limiting oocyst development through hemocyte participation, not only as effectors but also as signaling mediators. Nevertheless, further research is required to understand the communication mechanisms that exist between hemocytes and other mosquito organs.

During their development in the abluminal portion of the gut, oocysts make use of their extracellular cell wall (capsule) to evade the mosquito’s immune system [175]. The oocysts consume mosquito resources when undergoing sporogony, which involves a drastic increase in the size of the oocysts [180] along with the enlargement of the midgut basal lamina that envelops the oocysts. Interestingly, it has been reported that multiple blood feedings enhance oocyst growth and shorten the period of oocyst maturation [180–182]. Nevertheless, a recent study has shown that subsequent blood feedings reduce the oocyst number of rodent, but not human, *Plasmodium* parasites. This effect occurs because of the rupture of the basal lamina following blood meal-induced epithelium distention, which promotes direct exposure of the *P. berghei* oocysts to the mosquito’s complement-like system, an immune response that *P. falciparum* can evade [181].

Most of the previously mentioned mosquito-derived components of the oocyst capsule that favor oocyst maturation, such as laminin, collagen, LYSC1, and MMP1 [182–186], are highly expressed in hemocytes and can negatively regulate the complement-like system and melanization (Fig 3). Overall, these effects suggest that under certain conditions, hemocytes may be involved in oocyst protection. In addition, mosquito lipophorins, which are ingested by oocysts [187], can also enhance the survival of *P. berghei* oocysts [188].

In recent decades, several studies have revealed a plethora of mechanisms underlying the hemocyte-mediated immune response, with a main focus on their anti-plasmodial functions; however, their agonistic functions have received less attention. Furthermore, most of these studies have been performed using the rodent malaria parasite *P. berghei*, but it is well established that the mosquito’s immune system responds differently to *P. berghei* and the human malaria parasite *P. falciparum* [122,189,190]. To gain a better understanding of the role of hemocytes in *Plasmodium* infection, future studies should consider the compatibility of the experimental models (i.e., the *Plasmodium* and mosquito species used). Furthermore, to develop effective strategies for combating malaria by using genetically modified mosquitoes (GMOs), further studies are needed to focus on abolishing the mechanisms governing *Plasmodium’s* evasion of immunity.

### 4.3. Sporozoite invasion of the salivary gland

After their release into the hemolymph, the circulating sporozoites passively reach the basal lamina of the salivary glands (Fig 1) [191]. Salivary glands consist of three lobes attached to a common duct and are surrounded by epithelial cells arranged in a single layer [192]. Infecting this tissue provides the parasite access to the host, but it also represents a physical barrier to transmission [193]. Prior to the entry of the sporozoites into the salivary gland cavity, recognition and epithelial cell-crossing processes occur. These processes involve specific *Anopheles* receptors and other factors responsible for the initial attachment and invasion, as well as parasite proteins, including the surface proteins CSP, thrombospondin-related anonymous protein (TRAP), and membrane antigen/erythrocyte binding-like protein (MAEBL) [194].

CSP-mediated recognition involves salivary gland glycans and proteins such as CSP-binding protein (CSPBP). TRAP interacts with mosquito Saglin and salivary gland surface protein 1 (SGS1) and mediates directional migration [176]. Recent evidence shows that the *P. berghei* sporozoite protein, claudin-like apicomplexan microneme protein (CLAMP), is an essential participant in salivary gland traversal and sporozoite gliding motility, with the underlying mechanism involving the shedding of TRAP [195]. Also, the mosquito-encoded epithelial serine protease (ESP), expressed on the basal side of the epithelial cells, is essential for invasion of the sporozoite salivary gland [196]. Furthermore, RNAi-mediated silencing of a salivary gland-transcribed transmembrane glucose transporter (AGAP007752) results in significantly decreased sporozoite numbers, presumably because of its putative function as a sporozoite receptor [197]. Interestingly, a small peptide known as salivary gland and midgut binding peptide 1 (SM1) has been identified via a phage display peptide library and found to be distinctive in its ability to bind to epithelial receptors in the mosquito midgut and salivary gland, thereby interfering with parasite transmission [198].

### 4.4. Mosquito immune responses against sporozoites

**Fig 4:**
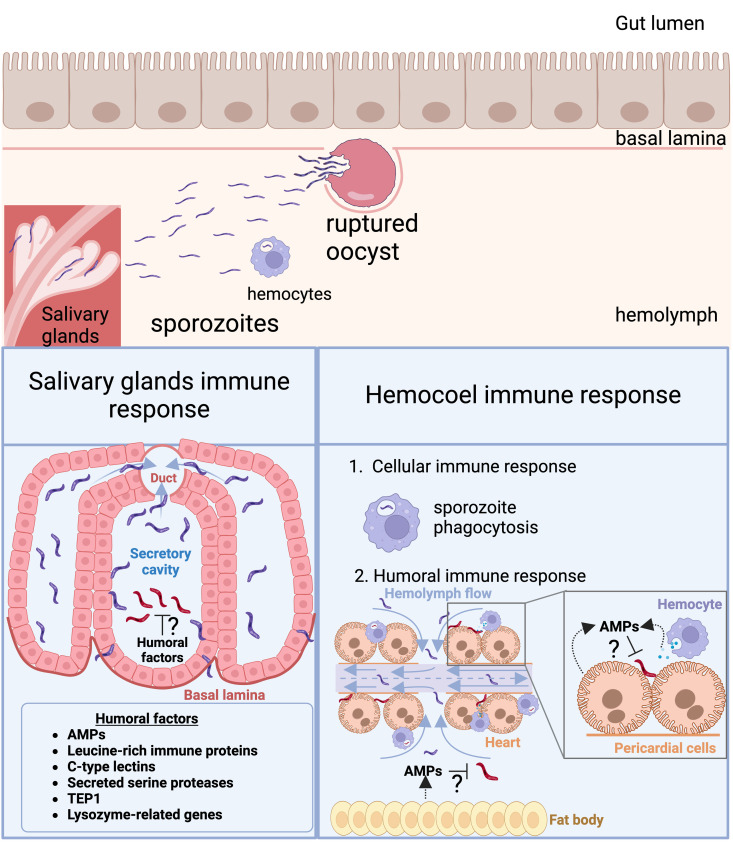
Mosquito immune responses against the sporozoite stage. Following the rupture of the mature oocysts, the released sporozoites become directly exposed to all the cellular and humoral immune responses in the hemolymph. Apart from hemocyte-mediated phagocytosis, sporozoites can also be subjected to fat body-secreted AMPs that can play an essential role in sporozoite lysis (right panel). After infection, the high hemolymph flow drives the hemocyte accumulation around the periostial region, promoting direct contact between circulating pathogens and hemocytes, and therefore phagocytosis. Also, AMPs are produced in pericardial cells and may be involved in the lysis of sporozoites that accumulate in the heart (right panel). AMPs, lysozyme-related genes, C-type lectins, serine proteases, leucine-rich immune proteins, and TEP1 are all expressed in the mosquito salivary glands; however, their effect on the sporozoite stage needs to be further investigated (left panel). Created in BioRender. Saab, S. (2025) https://BioRender.com/c22y701.

Sporozoites represent the parasite stage that experiences the greatest exposure to the hemolymph, since these final-stage parasites migrate to the heart and make use of the flow of the hemolymph as a transport medium to reach the salivary glands [199]. During this journey, they are entirely exposed to both humoral and cellular immune factors. As a result, in models involving *An. dirus-P. vivax* and *Ae. aegypti-P. gallinaceum*, only about 20% of the sporozoites produced in the oocysts actually reach the salivary glands, and the rest disappear in the hemolymph ([199,200]. Nevertheless, hemocyte-mediated immune responses, including phagocytosis, nodulation, encapsulation, and melanization in *An. gambiae*, *An. albimanus*, and *Ae. aegypti* only partially contribute to the elimination, respectively, of *P. berghei*, *P. vivax*, and *P. gallinaceum* sporozoites; instead, lysis is suggested as the major mechanism of their killing (Fig 4) [199,201–203].

The primary function of the mosquito heart is to pump the hemolymph around the mosquito’s body [204]. However, after infection, hemocytes accumulate around periostial regions, which are sites of high hemolymph flow, facilitating contact between circulating pathogens and hemocytes, and therefore phagocytosis (Fig 4) [201,205]. Interestingly, *P. berghei* sporozoites also accumulate in the periostial regions, where they undergo fragmentation [201]. Although hemocytes have been suggested to be the primary cell type involved in these processes, other cell types located in periostial regions, such as pericardial cells, could also play a significant role in parasite killing. Immune factors such as lysozyme and cecropin, produced in pericardial cells, could be involved in the lysis of pathogens accumulated in the heart [206–208]. In addition, other factors such as NO, ROS, and lysozymes are produced by pericardial cells and hemocytes, and they are implicated in establishing interactions between these cells [206,208–210]. However, whether these cells are involved directly or whether they cooperate with hemocytes in the elimination of sporozoites, as well as the mechanism(s) involved in this interplay, are largely unknown and warrant further study.

To date, only a few studies have highlighted the mosquito’s immune responses against sporozoites within the salivary glands. In *An. gambiae* and *An. coluzzii*, RT-PCR studies as well as serial analysis of gene expression (SAGE) and RNA-seq studies have revealed the induction of several immune-related genes by *P. berghei,* including AMPs, TEP1, and CTLMA2 [197,211,212]. A comparative transcriptomic analysis between *P. vivax*-infected and uninfected *An. dirus* salivary glands has demonstrated an elevated immune response in the infected glands, with up-regulation of several known AMPs as well as leucine-rich immune proteins and C-type lectins, among others (Fig 4) [213]. Moreover, several lysozyme-related genes, lectins, secreted serine proteases, and other uncharacterized secreted proteins have been identified in *Anopheles* salivary glands [214]. Remarkably, the transcriptional response at the organ level (salivary glands) after bacterial immune challenge is different from that induced systemically [215]. The studies just mentioned clearly indicate that *Anopheles* salivary glands not only serve as a physical barrier that has to be bypassed by *Plasmodium* and other microorganisms but also exert local immune activity that is induced after invasion/challenge. The relative contribution of the local salivary gland immune responses against sporozoites, where they remain up until the infectious bite occurs, has yet to be unveiled.

## 5. Sporozoite transmission to the host

As mosquitoes probe for blood, the infectious mature sporozoites exit the mosquito vector, accompanied by a mosquito saliva protein cocktail co-injected into the host [216]. Apart from facilitating the blood meal through their immunomodulatory, coagulation, anti-inflammatory, anti-hemostatic, and vasodilatory functions, a number of these sialome factors also affect the sporozoites’ transmission and infectivity in the vertebrate recipients [217,218]. Remarkably, the number of sporozoites injected into the mammalian host is by far smaller than the number of parasites found in the salivary glands [193], yet it correlates with the initial load [219]. Sporozoite infectivity in the host is initially locally affected either directly by mosquito factors that bind to the parasite [220] or indirectly by the initial dermal immune reaction that is prompted in response to the injected parasite, the mosquito proteins, and the mechanical damage caused by the bite [221]. Mosquito GILT, named after its similarity to human gamma interferon-inducible thiol reductase, binds to sporozoites and decreases their motility in mice [220], while sporozoite-associated mosquito saliva protein 1 (SAMSP1) positively affects sporozoite entry into host cells, thereby facilitating sporozoite infectivity [222]. Although the effectiveness of the host’s immune response against mosquito factors in influencing the subsequent parasite infectivity is controversial [223,224], accumulating recent evidence has demonstrated that some of these factors have immunomodulatory activity, with the potential to be harnessed as vaccine targets [216,225,226]. In this context, experiments on mice involving immunization with saliva-derived antiserum have resulted in a reduction in host-parasite infection as a result of decreased vascular permeability, mostly attributed to the secreted salivary gland protein AgTRIO [223]; in contrast, the sporozoite-associated factor (SAP) seems to affect sporozoite infectivity by modulating the host’s inflammatory responses [225]. Also, the saliva microRNA (miRNA) repertoire of hematophagous insects has been proposed to contribute to antiparasitic host responses [227]. Interestingly, the *An. coluzzii* miRNA patterns are distinct for the saliva and the salivary glands, with some of them being identical to human miRNAs with immune-regulatory roles [227,228]. However, as far as mosquitoes are concerned, the effect of these non-immunogenic factors during rapid blood-meal acquisition remains elusive and favors hypotheses regarding host immune regulation with long-term evolutionary advantages to the host [227]. Finally, the presence of the microbiota in salivary glands and saliva, and the transfer of specific bacteria during blood meals into mammalian hosts highlight additional saliva factors that could potentially influence *Plasmodium* transmission [229].

## 6. A possible role for salivary gland proteins in host-to-vector transmission

The mosquito salivary proteins blended into the blood meal have recently been shown to play a crucial role in facilitating parasitic infection of the mosquito gut. For example, the salivary mosquito protein known as Saglin was shown to mediate *Plasmodium* infection in the mosquito gut; however, the mode of action of this protein is still not known [230]. In addition to Saglin, the *Anopheles* salivary apyrase has been shown to facilitate fibrinolysis, thus promoting *Plasmodium* parasite infection through the activation of the tissue plasminogen activator in the blood bolus [231,232]. These studies highlight the potential of such mosquito salivary proteins to be harnessed as malaria transmission-blocking targets, either through knocking out such factors or exploiting salivary gland promoters to drive the expression of antiparasitic effectors. For instance, the use of transgenic *Anopheles* mosquitoes capable of targeting the fibrinolytic system by expressing human plasminogen activator inhibitor 1 (PAI-1) has been successful in reducing *Plasmodium* transmission to mosquitoes [233].

## 7. Mosquito immune memory and adaptive ability

Despite their lack of adaptive immunity, mosquitoes possess an innate immune memory that protects them against a repeated exposure to the same pathogen during a second encounter, a phenomenon known as immune priming [234]. Mosquitoes that were previously infected with *P. berghei* or *P. falciparum* have shown a reduction in the number of oocysts during the second exposure to the same parasite [235–238]. The mechanisms of immune memory in mosquitoes are not clear; some mechanisms have been proposed that involve hemocytes and midgut epithelial cell immune responses. One of these mechanisms posits direct contact of the microbiome with the epithelial cells when the ookinetes invade the midgut epithelium, triggering a systemic immune response that involves hemocyte differentiation [237,238]. This response produces an increase in the granulocyte subpopulation, changes in hemocyte morphology, and an overexpression of anti-plasmodial genes, including TEP1 and LRIM1. Thus, the mosquito triggers an enhanced immune response against the parasite during the second encounter [238]. Furthermore, midguts from mosquitoes infected with *Plasmodium* have shown high rates of DNA synthesis [235,236], and the inhibition of this process results in the abolition of protection during the second encounter with the parasite [236]. It has been suggested that some immune genes could be expanded as a result of endoreplication to increase their expression during a second encounter [236].

Although mosquitoes’ repertoire of pattern recognition receptors (PRRs) is limited, they provide a broad recognition for most pathogens. In addition to classic PRRs such as lectins and others, insects encode an immunoglobulin superfamily member called Down syndrome cell adhesion molecule (DSCAM) [234]. Through alternative splicing, DSCAM can produce more than 30,000 isoforms [239]. In mosquitoes, the splicing of some DSCAM isoforms is favored, depending on the pathogen, and this repertoire correlates with the affinity and activity against each pathogen [240]. Some DSCAM isoforms can discriminate between *Plasmodium* species and provide species-specific protection; however, it is not clear whether they interact with other effector molecules to kill the parasite [240,241]. Furthermore, it is unclear whether the isoform repertoire is maintained for a sufficient time after the immune challenges to provide immune memory. Innate immune memory in insects is probably the consequence of many mechanisms working together, which need to be deeply explored.

## 8. Conclusion

A great body of literature pinpoints the critical processes that facilitate *Plasmodium* invasion of the mosquito vector, yet many questions remain open and require deeper exploration to allow us to understand more thoroughly how various immune responses are coordinated to eliminate the invading parasites. However, the capacity of *Plasmodium* to overcome the well-orchestrated immune responses by the mosquito necessitates a focus of current research on developing transmission-blocking strategies for reducing mosquito vector competence. Recent efforts have been directed at generating genetically modified mosquitoes that are resistant to *Plasmodium* [79,112,141,242]. Other recent advances in dissecting the parasite’s complex life cycle in the mosquito vector have helped to identify potential TBV targets. However, to eradicate malaria, such strategies must be combined with an effective malaria vaccine and preventive drugs.
